# Impact of an Anticoagulation Management Service–Led Annual Review on Antiplatelet Use and Gastrointestinal Bleeding Prophylaxis in Ambulatory Patients on Background Direct Oral Anticoagulants

**DOI:** 10.1007/s11239-026-03283-7

**Published:** 2026-04-29

**Authors:** Chau Hoang, Peter Collins, David DeiCicchi, Ewan McNicol, Devan Hawkins, Kathy Zaiken

**Affiliations:** 1 Boston Medical Center Brighton, 11 Nevins St, Boston, MA 02135 USA; 2 Anticoagulation Management Service, Atrius Health, Watertown, MA USA; 3 Massachusetts College of Pharmacy and Health Sciences, Boston, MA USA

**Keywords:** Direct oral anticoagulants, Aantiplatelet, Aaspirin, Dual antithrombotic therapy, Triple antithrombotic therapy, Deprescribing, Proton pump inhibitor, Centralized anticoagulation services, Aanticoagulation stewardship

## Abstract

Patients on long-term anticoagulation face increased bleeding risk when antiplatelets are co-prescribed, especially upper gastrointestinal (GI) bleeding. Guidelines recommend limiting antiplatelet use to highly select patients on background anticoagulants and promoting GI prophylaxis with proton pump inhibitors (PPIs) for those requiring dual antithrombotic therapy. To assess the impact of an anticoagulation management service (AMS)-led intervention on reducing excess antiplatelet use and increasing PPI prescribing in patients who require continued antiplatelet therapy. This study of retrospective chart review included adults (≥ 18 years) on long-term direct oral anticoagulant (DOAC) therapy from October 2023 to September 2024. The intervention group included AMS-enrolled patients who received structured annual anticoagulation review. The control group consisted of DOAC patients receiving usual care outside AMS. The AMS annual review aims to optimize antithrombotic therapy by deprescribing unnecessary antiplatelets and initiating PPIs when clinically indicated. Of 8,462 eligible patients, 3,125 were in the AMS group and 5,337 in the non-AMS group. Antiplatelet use was significantly lower in AMS patients at baseline (9% vs. 15%) and study end (7% vs. 16%) (*p* < 0.0001). PPI use was significantly higher in AMS patients at both time points (50% vs. 30% for baseline, 67% vs. 38% at the end of study) (*p* < 0.0001). Acceptance rates for AMS recommendations were 18.8% for antiplatelet discontinuation and 33% for PPI initiation. AMS-led interventions effectively reduced concurrent antiplatelet use in patients on background DOAC and increased PPI prescribing in those on dual therapy, demonstrating the value of centralized anticoagulation services in primary care.

**Graphical Abstract:**

**Figure:**
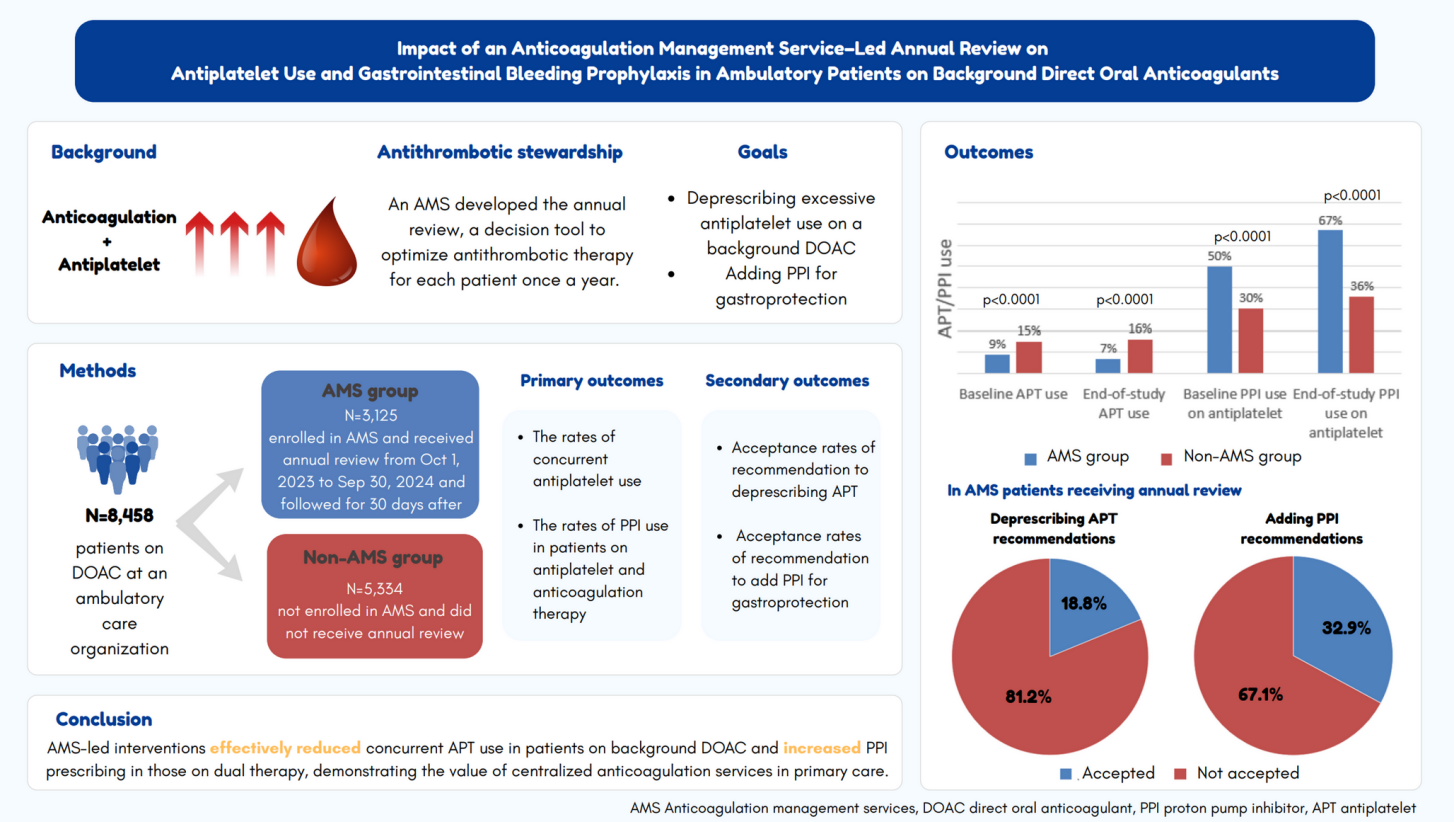
Overview of the AMS-led annual review intervention and its impact on deprescribing antiplatelet therapy and adding PPI for gastroprotection in patients receiving DOACs. AMS = anticoagulation management service; PPI = proton pump inhibitor; DOAC = direct oral anticoagulant.

**Supplementary Information:**

The online version contains supplementary material available at 10.1007/s11239-026-03283-7.

## Key Points

Patients on long-term anticoagulation are at increased risk of bleeding when antiplatelets are co-prescribed, highlighting the need for careful medication review via a centralized anticoagulation service (AMS).

An AMS-led annual review effectively reduced excessive antiplatelet use in patients on direct oral anticoagulants.

AMS interventions increased proton pump inhibitor prescribing for gastric bleeding prophylaxis among patients requiring dual antithrombotic therapy.

Further studies are needed to evaluate the clinical outcomes and cost-effectiveness of AMS-led interventions in preventing bleeding while preserving cardiovascular protection.

**Supplementary Information:**

The online version contains supplementary material available at 10.1007/s11239-026-03283-7.

## Introduction

Patients with atrial fibrillation (AF) and, in some cases, venous thromboembolism require long-term anticoagulation to prevent serious systemic thrombotic complications. For the last decade, anticoagulants have accounted for the highest rate of adverse drug events leading to emergency department visits compared to any other class of medications, with nearly 80% of cases due to hemorrhage [1]. Many patients with AF and VTE often also have comorbid conditions that are indications for antiplatelet use. For example, half of patients with AF also have coronary artery disease (CAD) and requires careful assessment of antithrombotic regimens [2]. The addition of single antiplatelet therapy (SAPT) to oral anticoagulation (OAC) increases the risk of nonfatal and fatal bleeding by 2-fold to-3 fold while the addition of dual antiplatelet, known as triple therapy, increases the risk by nearly 4-fold [3]. Therefore, effective management requires balance between preserving antithrombotic efficacy and minimizing bleeding risk.

The benefits of antiplatelet therapy for the prevention of cardiovascular events are well recognized. However, antiplatelet agents should be used with caution in combination with anticoagulants and generally only be reserved for high-risk patients, such as those with recent acute coronary syndrome (ACS) or percutaneous coronary intervention (PCI). In many cases the risks of combining antiplatelet and anticoagulant therapy may outweigh the benefits, particularly in patients who are not at high cardiovascular risk, such as those with chronic coronary disease (CCD) or when being used for primary prevention purposes. In patients with AF undergoing PCI and requiring triple antithrombotic therapy, national and international cardiovascular societies recommend limiting the duration of triple therapy to 1–4 weeks following PCI to minimize bleeding risk [4–9]. This is supported by current evidence indicating that triple therapy after 30 days increases the risk of bleeding without reducing ischemic events [10–13]. A more recent national guideline on the management of acute coronary syndromes recommends using the Academic Research Consortium for High Bleeding Risk (ARC-HBR) [14] criteria to identify patients at high bleeding risk following PCI. The tool describes long-term use of oral anticoagulation as one of the major criteria that independently determines patients as being high bleeding risk, which supports the short duration of triple antithrombotic of 1 to 4 weeks post-discharge [15]. For patients with history of ACS and an indication for anticoagulation, current guidelines recommend limiting dual antithrombotic therapy to a maximum duration of 6–12 months following the ACS event or PCI [4–9]. For those patients who are not at high ischemic risk, duration of clopidogrel may be shortened to 3 months per recommendation from the 2024 ESC guideline [7]. Data from RCTs and meta-analysis demonstrated that omitting antiplatelet beyond 6 to 12 months after PCI or CABG significantly reduced risk of bleeding without significant difference in ischemic events [16–18]. Despite a paucity of data, some guidelines have extrapolated dual and triple antithrombotic therapy guidance to patients on anticoagulation for other indications such as VTE, and to patients on antiplatelets for alternative indications such as stable peripheral artery disease (PAD) and cerebral vascular disease [4]. These nuances underscore the importance of individualized risk assessment to mitigate bleeding risk while preserving efficacy in patients at high risk for ischemic cardiovascular disease.

Upper gastrointestinal bleeding (UGIB) is the most common major bleeding adverse event associated with combined antiplatelet and anticoagulant use, with a disproportionate increase in rates compared to other types of bleeding [19]. Unless contraindicated, direct oral anticoagulants (DOACs) are generally preferred over warfarin given the lower risk of intracranial hemorrhage and practical advantages. However, the use of DOACs is associated with an increased risk of UGIB compared to warfarin as shown in pivotal trials for non-valvular atrial fibrillation (NVAF), with apixaban being the only exception [20–23]. These findings highlight the importance of gastroprotection strategies in patients receiving combined antithrombotic therapy. Proton pump inhibitors (PPIs) have been shown in cohort studies to reduce the incidence of UGIB when used during treatment with warfarin or DOACs in patients with high risk of bleeding [24, 25], and are recommended by national cardiovascular and gastroenterology societies for patients receiving dual antithrombotic therapy [15, 26, 27]. Histamine-2 receptor antagonists (H2RAs) modestly reduce the risk of UGIB in patients on antithrombotic therapy compared to placebo and remain a reasonable alternative to PPI [27].

Based on current evidence and guidance from leading professional societies, the Atrius Health Anticoagulation Management Services (AMS) developed internal guidelines and practice protocols to guide management of antithrombotic therapy, including identifying and intervening on excessive antiplatelet use and the addition of a PPI for gastric bleeding prophylaxis for patients that require combined therapy. In October 2023, AMS launched an initiative to optimize anticoagulation for all enrollees via an annual comprehensive review of antithrombotic therapy. This review assesses the indication and duration of therapy for anticoagulation, appropriateness of anticoagulant agent, quality of anticoagulation (e.g., warfarin time in target range or medication adherence), co-prescribed antiplatelets, and risk of both thrombosis and bleeding. This study aims to evaluate the impact of this initiative on reducing unnecessary antiplatelet use and increasing rates of PPI prescribing for gastroprotection in appropriate patients receiving DOACs within an ambulatory care setting.

## Methods

### Study Design

This observational study was designed as a retrospective chart review of data from the electronic medical record (EMR). The study was approved by the Institutional Review Boards at Massachusetts College of Pharmacy and Health Sciences and Atrius Health (Approval Number 2024202527) with a waiver of informed consent. Patients were included if they were 18 or older with documented long-term DOAC use from October 2023 to September 2024. The treatment group (“the AMS group”) consisted of patients managed under collaborative drug therapy management (CDTM) and nursing protocols within the centralized Atrius Health AMS who underwent annual review between October 2023 and September 2024. The annual review is a process developed by AMS to optimize antithrombotic therapy, including reducing excess antiplatelet use and increasing PPI use for prevention of UGIB in high-risk patients. Continued use of H2RA was allowed if use was stable at baseline. The usual care group (“the non-AMS group”) consisted of patients taking DOACs that were not enrolled in AMS and managed instead by their prescribing team. Patients were excluded if they were taking rivaroxaban 2.5 mg twice daily for the reduction of major cardiovascular events in CAD or major thrombotic vascular events in peripheral artery disease, since this indication requires combination therapy with aspirin 81 mg daily.

To assess bleeding risk and recommend an individualized antithrombotic treatment plan in the annual review, AMS may utilize HAS-BLED and ARC-HBR bleeding risk scoring systems to further stratify bleeding risk if clinically relevant [14, 28]. Details of the AMS clinical guideline and practice protocols pertaining to the annual review and concurrent antiplatelet and anticoagulation therapy are provided in the supplementary appendix. Any proposed changes to antithrombotic regimens are communicated to the referring physician or care team before implementation.

### Data Collection

Patients were identified from the EMR based on the inclusion and exclusion criteria listed above. Baseline data including age, sex, specific DOAC agent and indications for use, specific antiplatelet agent and indications for use, dual antiplatelet use, and PPI or H2RA use were collected (Table 1). Use of medications such as DOACs, antiplatelets, PPIs, and H2RAs was determined based on the current medication list at different points in time and their respective start dates and end dates. Medications were considered active at baseline if the start date of the medication order was before October 1, 2023, and the end date was after October 1, 2023 (or if no end date specified). Similarly, end-of-study use of medications was determined if the start date of the medication order was before October 31, 2024. The end-of-study date to capture medication use was extended by one month to allow time for clinician response to AMS recommendations if the annual review was conducted towards the end of September 2024.

### Outcomes

The primary outcome compared the rates of background antiplatelet use and rates of PPI use for gastric prophylaxis in patients on dual antithrombotic therapy between AMS patients who underwent the annual review and non-AMS patients. The secondary outcomes assessed clinician acceptance rates of recommendations made by AMS to discontinue antiplatelets or to start PPIs during the annual review. AMS recommendations were documented in the patient’s electronic health record and included an individualized assessment of antithrombotic therapy and supporting rationale. Recommendations were communicated to the referring physician (primary care provider or applicable specialty clinician) via the electronic health record messaging. AMS followed up with providers via the electronic health record messaging after four weeks if no response had been received. Additional details regarding communication with external physicians are provided in the Supplementary Appendix.

### Statistical Analysis

A sample size calculation was performed based on a preliminary review of data in the system, which found that about 13% of the patients enrolled in AMS were using antiplatelets concurrently with DOACs. A minimum sample size of 240, with 120 patients in each of the study groups, would be needed to detect at least a 30% relative decline in antiplatelet use with a power of 80% and an alpha of 0.05.

For AMS and non-AMS patients, we separately calculated the frequency of the following variables: sex, baseline DOAC use, indication for DOAC, baseline antiplatelet use, indication for antiplatelet use, and baseline PPI or H2RA use with antiplatelets. A two-sided Fisher’s exact test was used to compare the distribution of these variables between the two groups. Additionally, the mean age and associated standard deviations were calculated for both groups. Means were compared using a two-sample t-test.

We calculated the percentage of patients with baseline and end-of-study antiplatelet use for both study groups. Among patients who were taking a concurrent antiplatelet on background DOACs, we calculated the percentage of baseline and end-of-study PPI use in both groups. All these frequencies were compared using Fisher’s exact test.

Among patients enrolled in AMS who underwent the annual review, rates of acceptance were calculated using the total number of accepted recommendations and the total number of overall recommendations made during the 2023–2024 annual review for the study populations. For patients receiving concurrent antiplatelet therapy, a recommendation can be made to suggest discontinuation of antiplatelets or to recommend adding PPI therapy for gastric bleeding prophylaxis if dual antithrombotic therapy is indicated. A recommendation is considered accepted if action, including stopping antiplatelets or adding a PPI, is made within four weeks of the annual review date. Finally, the antiplatelet deprescribing acceptance rates were stratified according to the indications for antiplatelet use (i.e., history of ACS or CCD, cerebrovascular disease, and PAD) and Fisher’s exact test was used to examine differences in acceptance across these categories.

## Results

### Baseline Characteristics

**Fig. 1:**
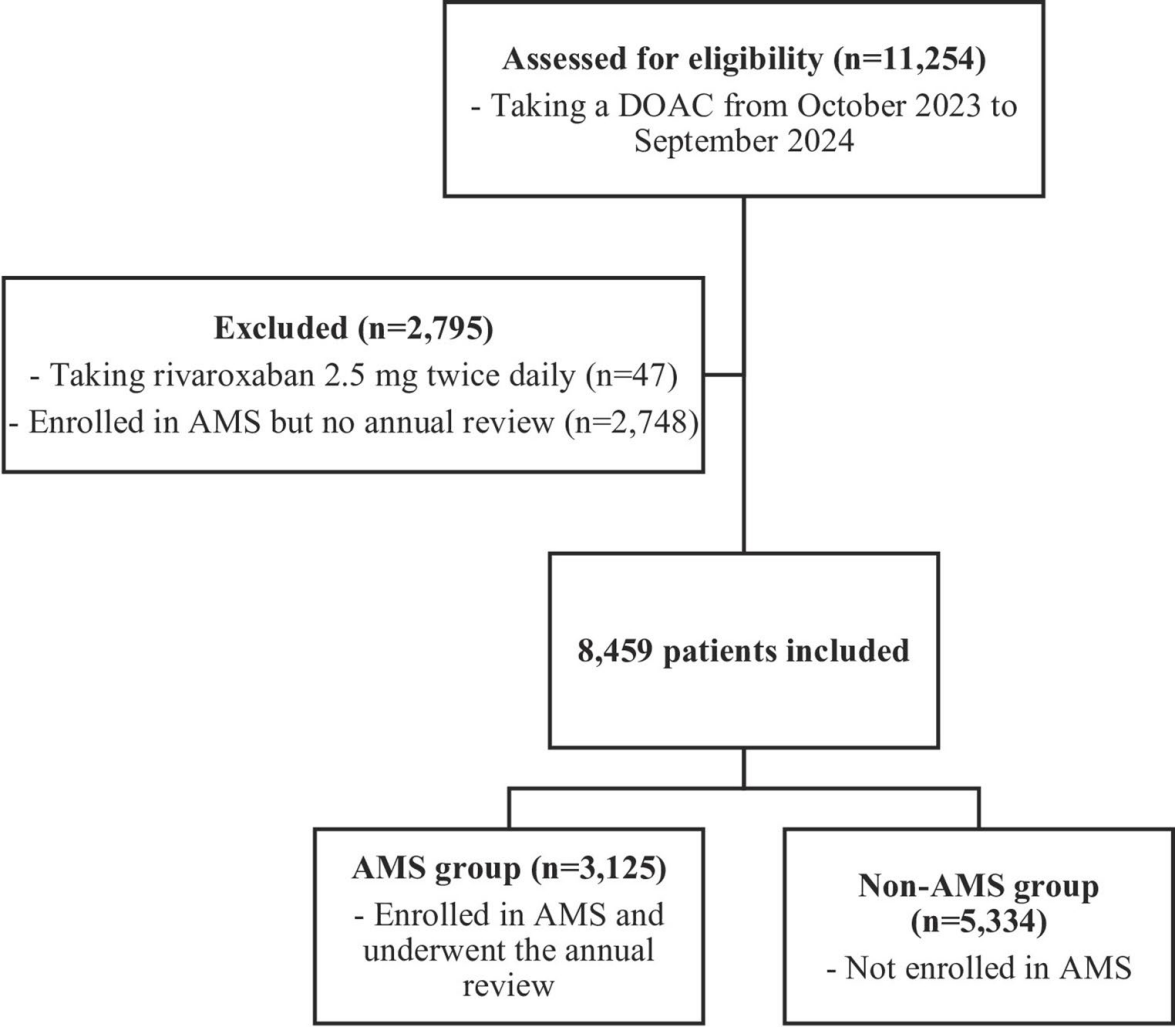
Patient flow diagram. Flowchart depicting patient identification, inclusion and exclusion criteria, and final study cohort

Between October 2023 and September 2024, a total of 8,459 patients met all inclusion criteria and were included in the study. A total of 2,748 patients enrolled in AMS did not receive an annual review and were excluded, which could be due to death, disenrollment before annual reviews were due, or being enrolled in AMS within a year of the study end date and thus not due for their first annual review during study period. Patients who were deceased or discharged but had received an annual review were eligible to be included in the study as AMS patients. A total of 47 patients were excluded as they were taking rivaroxaban 2.5 mg twice daily for peripheral artery disease or CAD to reduce risk of cardiovascular events (Fig. 1).

As shown in Table 1, more than half of the patients were male, and there was a significant difference in the sex distribution between the AMS and non-AMS groups, (58% in AMS vs. 55% in non-AMS, *p* = 0.0134). Additionally, there was a significant difference in baseline DOAC agent use between groups, with a larger percentage of non-AMS patients taking apixaban (81% vs. 75%) and a larger proportion of AMS patients on rivaroxaban (24% vs. 19%). The overall distribution difference was statistically significant (*p* < 0.0001). The majority of patients were taking a DOAC for NVAF (74% in AMS vs. 67% in non-AMS; *p* < 0.0001 for overall distribution). At baseline, antiplatelet use on a background DOAC differed significantly between the two groups and was higher in the non-AMS group (15% vs. 9%; *p* < 0.0001). No significant difference in dual antiplatelet use was found between the two groups at baseline (1% vs. 3%; *p* = 0.1136). All three dual antiplatelet therapies (DAPT) in the AMS group were combination therapy of clopidogrel and aspirin. The most common DAPT in the non-AMS group on a background anticoagulant was also clopidogrel and aspirin (20 out of 23, or 87%). There were only two non-AMS patients taking ticagrelor with aspirin, and one patient taking the combination of prasugrel and aspirin. “Other” was if patients had no active FDA-approved indications for antiplatelet or DOAC, or if patient did not have a diagnosis listed in the medical history on the electronic health system at the time of data collection. PPI use was significantly higher in the AMS group compared to the non-AMS group among those who were taking both antiplatelets and DOACs at baseline (50% vs. 29%; *p* < 0.0001). H2RA use on antiplatelets did not differ between the two study groups (9% vs. 8%; *p* = 0.9008).

**Table 1:** Baseline characteristics of patients

Characteristic	AMS group (*n* = 3125)	Non-AMS group (*n* = 5334)	*P*-value
*Sex (%)*			0.0134
Female	1317 (42%)	2396 (45%)	
Male	1808 (58%)	2937 (55%)	
*Age (years)*	74.3 ± 11.6	73.4 ± 13.2	0.0016
Range	22–106	18–104	
*Baseline DOAC use*			< 0.0001
Apixaban	2352 (75%)	4303 (81%)	
Rivaroxaban	751 (24%)	992 (19%)	
Dabigatran	20 (1%)	37 (1%)	
Edoxaban	2 (0%)	2 (0%)	
*Indications for DOAC* ^¥^			< 0.0001
NVAF	2321 (74%)	3569 (67%)	
DVT	363 (12%)	478 (9%)	
PE	367 (12%)	466 (9%)	
Other VTE	35 (1%)	82 (2%)	
Other^¶^	264 (8%)	1002 (19%)	
*Baseline antiplatelet use*	280 (9%)	818 (15%)	< 0.0001
Aspirin	256 (91%)	728 (89%)	
Aspirin/dipyridamole	0 (0%)	1 (0%)	
Clopidogrel	26 (9%)	108 (13%)	
Prasugrel	0 (0%)	1 (0%)	
Ticagrelor	1 (0%)	3 (0%)	
*Indications for antiplatelet use* ^¥^			0.0198
History of ACS or Chronic coronary disease^€^	163 (58%)	420 (51%)	
Cerebral vascular disease£	126 (45%)	307 (38%)	
PAD	49 (18%)	116 (14%)	
Other^¶^	72 (26%)	280 (34%)	
Baseline dual antiplatelet use	3 (1%)	23 (3%)	0.1136
Baseline PPI use for patients on single antiplatelet therapy	141 (50%)	241 (29%)	< 0.0001
Baseline PPI use for patients on dual antiplatelet therapy	2 (67%)*	10 (43%)*	
Baseline H2RA use for patients on single antiplatelet therapy	24 (9%)	68 (8%)	0.9008
Baseline H2RA use for patients on dual antiplatelet therapy	0 (0%)*	3 (13%)*	

NVAF denotes non-valvular atrial fibrillation, DVT deep vein thrombosis, PE pulmonary embolism, VTE venous thromboembolism, ACS acute coronary syndrome, CCD chronic coronary disease, PAD peripheral artery disease.

^¥^Patient can have multiple concurrent indications for DOAC or antiplatelet use.

^€^History of ACS includes patient with STEMI, NSTEMI, and unstable angina with or without coronary revascularization procedures (i.e., CABG, PCI), CCD includes patients with a history of ACS for more than one year earlier who have remained free of recurrent ACS.

^£^Cerebral vascular disease includes history of cerebrovascular accident (CVA)/transient ischemic attack (TIA), carotid artery stenosis, and history of carotid artery stenting.

^*^Percentage represents patients receiving PPI or H2RA therapy among those on dual antiplatelet therapy (DAPT).

^¶^Other includes patients without an active FDA-approved indications for DOAC or antiplatelet use, or without a diagnosis listed in the medical history on the electronic health system.

### Primary Outcomes

The rate of antiplatelet use in the AMS group decreased from 9% to 7% during the study period while the rate in the non-AMS group increased from baseline from 15% to 16% (Table 2). Regardless of the opposing trends in use, antiplatelet use was consistently and significantly higher among the non-AMS group compared to the AMS group throughout the study period (9% vs. 15% for baseline, 7% vs. 16% at the end of study; *p* < 0.0001 for both). End-of-study dual antiplatelet use was numerically higher in the non-AMS group but did not differ significantly compared to the AMS group (4% vs. 3%; *p* = 0.1227). PPI use for patients on dual antithrombotic therapy increased in both groups over time; however, the AMS group had significantly higher rates of PPI use both at baseline (50% vs. 29%; *p* < 0.0001) and at the end of the study (67% vs. 36%; *p* < 0.0001), and also saw a larger relative rate increase over time (34% increase compared to a 20% increase in the non-AMS group).

**Table 2:** Antiplatelet and PPI use at baseline and study end

	AMS *N* (%)	Non-AMS *N* (%)	*P*-value
*Antiplatelet use*			
Baseline antiplatelet use	280 (9%)	818 (15%)	< 0.0001
End-of-study antiplatelet use	231 (7%)	867 (16%)	< 0.0001
End-of-study dual antiplatelet use*	5 (2%)	32 (4%)	0.1227
*PPI use in patients on dual antithrombotic therapy*			
Baseline PPI use	141 (50%)	241 (29%)	< 0.0001
End-of-study PPI use	154 (67%)	315 (36%)	< 0.0001

*Percentage calculated among total antiplatelet users at study end.

### Secondary Outcomes

In total, AMS made 387 recommendations (or 12%) regarding deprescribing antiplatelet or initiating PPI in the 2023–2024 annual review cycle. AMS made 308 recommendations to clinicians to deprescribe antiplatelets during this annual review, of which 18.8% (or 58) were accepted (Table 3). Among 79 recommendations to initiate PPIs in patients on dual antithrombotic therapy, 33% (or 26) were accepted, as evidenced by PPI initiation within four weeks of the annual review date. Twenty-five patients received omeprazole while only one received pantoprazole for gastric prophylaxis as a result.

**Table 3:** Antiplatelet and PPI 4-week acceptance rate in AMS group

	Total recommendations made (*n*)	Total accepted recommendations (*n*)	Acceptance rate (%)
Deprescribing antiplatelet	308	58	18.8%
Adding PPI	79	26	32.9%

### Post Hoc Analysis

A post hoc analysis was conducted among patients in the AMS group for whom antiplatelet deprescribing was recommended based on individualized assessment of thrombotic and bleeding risk. The study population was stratified according to antiplatelet therapy indication to account for factors that may influence acceptance of AMS recommendations. No statistically significant differences were observed between patients with accepted versus non-accepted recommendations across antiplatelet therapy indications (Table 4). Although patients with a history of ACS or CCD showed a numerically higher rate of recommendation acceptance (67.2% accepted vs. 55.2% not accepted), this association was not statistically significant (*p* = 0.1911).

**Table 4:** Acceptance of AMS Antiplatelet Deprescribing Recommendations Stratified by Indication (Post hoc Analysis)

	Recommendations accepted*N* (%)	Recommendations not accepted*N* (%)	*P*-value
Total	58 (100%)	250 (100%)	
History of ACS or CCD Yes No	39 (67.2%) 19 (32.8%)	138 (55.2%) 112 (44.8%)	0.1911
Cerebral vascular disease Yes No	26 (44.8%) 32 (55.2%)	113 (45.2%) 137 (54.8%)	1
PAD Yes No	7 (12.1%) 51 (87.9%)	32 (12.8%) 218 (87.2%)	0.586

ACS acute coronary syndrome, CCD chronic coronary disease, PAD peripheral artery disease.

## Discussion

The results of this study showed that concurrent antiplatelet use was reduced at end of study in the AMS group while usage increased in the usual care group. PPI use in patients on both antiplatelet and anticoagulant agents increased in both groups at the end of the study, but the AMS group saw a greater net increase compared to usual care. The AMS group has significantly lower concurrent antiplatelet use and significantly greater PPI use for UGIB prophylaxis compared to the non-AMS group both at baseline and at the end of the study.

The difference in concurrent antiplatelet use at baseline between AMS and non-AMS patients can be attributed to the systematic approach the AMS team employs to optimize DOAC management. Patients managed by AMS undergo a standardized process the time of enrollment that includes a clinical review of their anticoagulation and related therapies, along with periodic outreach to support safe and effective use through monitoring, education, and ad hoc interventions. Notably, a substantial proportion of patients in the study population had no documented indication for DOAC therapy or were receiving off-label DOAC use, as reflected by the percentage categorized as “Other” in Table 1. However, although AMS also evaluates the appropriateness of DOAC indication and duration during annual reviews, this study focused specifically on optimizing antiplatelet therapy and gastric bleeding prophylaxis in patients receiving background DOAC therapy and therefore did not report recommendations or outcomes related to DOAC use. For the secondary outcome, the total number of recommendations made to deprescribe antiplatelets is higher than the number of patients on antiplatelets at baseline and study end. The study only captured concurrent antiplatelet use at the beginning and end of the study period. As a result, patients who started antiplatelet therapy in either arm during the study period were not captured but could still have recommendations to deprescribe antiplatelets. AMS patients may have experienced changes in clinical status after their annual review and may have discontinued or restarted antiplatelet or PPI therapy before the end of the study period. Therefore, the decrease in antiplatelet use observed at the end of the study represents a net change in overall antiplatelet use within the study population. Interestingly, patients with a history of ACS or CCD had a higher rate of antiplatelet deprescribing recommendation accepted; however, the difference was not statistically significant. This observation was unexpected, as clinicians tend to exercise greater caution when deescalating antiplatelet therapy in patients with established coronary disease.

The results of our study are similar to prior studies that evaluate the impact of a pharmacist-led protocol or initiative on deprescribing potentially inappropriate antiplatelet use in patients taking long-term anticoagulation. Meador et al. reported the success of a systematic antithrombosis stewardship intervention led by pharmacists in deprescribing concomitant antiplatelet in an outpatient anticoagulation clinic system in Albuquerque, New Mexico [29]. The de-escalation protocol allowed pharmacists to discontinue antiplatelet therapy or communicate with providers to further discuss discontinuation. The result was 45 (93%) discontinuations among patients whose use was deemed inappropriate, or a total antiplatelet use declined from 30% to 25% among the total of 875 participants who were taking anticoagulant long-term. Another similar study was conducted to assess the effectiveness of pharmacist-led quality improvement initiative in an outpatient anticoagulation clinic of a community hospital located in Toledo, Ohio [30]. This study also reported 33% concurrent aspirin use at baseline. The initiative successfully identified 22 patients whose aspirin use was deemed inappropriate and reported an acceptance rate of 45% when aspirin deprescribing was recommended to clinicians. A quality improvement observational study conducted in six outpatient anticoagulation clinics in Michigan demonstrated reduction in major bleeding events when aspirin was deprescribed among patients on warfarin for AF and/or VTE (0.31% vs. 0.21%, *p* = 0.03 for difference in slope before and after the intervention) [31]. A 2025 meta-analysis by Rashedi et al. pooled efficacy and safety outcomes from four RCTs including patients with AF receiving OAC with or without concomitant antiplatelet therapy [18]. Included patients were at least 6 months post-PCI or CABG for stable CAD or at least one year post-PCI or CABG for ACS. The results demonstrated a significant reduction in major bleeding with OAC monotherapy compared to OAC plus SAPT (3.3% vs. 5.7%; HR: 0.59; 95% CI: 0.44–0.79; *p* < 0.001; I^2^ = 0%). OAC monotherapy was also associated with a lower risk of major or clinically relevant nonmajor bleeding (10.0% vs. 18.4%; HR: 0.53; 95% CI: 0.44–0.63; *p* < 0.001; I^2^ = 58%) based on data from three of the included trials. Since then, additional studies have reported no clear clinical benefit from continuing antiplatelet therapy in patients requiring long-term oral anticoagulation, while observing an increase in major bleeding risk and a potential association with higher rates of cardiovascular events and mortality [32, 33].

A major difference in our study design is that these studies did not compare rates of use to a control group receiving usual care along with major differences in baseline antiplatelet use. Overall, the rates of concurrent antiplatelet use in our population, regardless of AMS enrollment status, were substantially lower than those reported in the three studies above. This difference may reflect institution-specific prescribing patterns, increased adherence to current evidence-based guidelines, or a broader downward trend in combined antiplatelet and anticoagulant therapy over time [34]. Additionally, aspirin is available over-the-counter, its use may have been underreported in the non-AMS group, where no formal verification process existed, unlike the AMS group where it is standard practice to inquire about antiplatelet use on enrollment and document on the medication list. The low rates of antiplatelet use in the AMS group likely reflect formal antiplatelet use guidance developed by AMS and standard antiplatelet use assessments that were completed on enrollment prior to the start of the study period. Similarly, gastroprotective measures are assessed at AMS enrollment and since AMS only recommends PPIs, this process likely contributed to the higher PPI use observed at the start of the study period compared to the non-AMS group while the rates of H2RA use were similar.

It is AMS practice to coordinate non-anticoagulant clinical decisions, such as stopping antiplatelets or prescribing PPIs, with key stakeholders in the patients care and therefore we require consultation with the primary care provider and/or specialist prior to enacting these changes. The acceptance rate of antiplatelet deprescribing in our study was lower than that reported in prior studies [29–31]. Since it is standard for AMS to address antiplatelet use on enrollment, subsequent deprescribing decisions may have involved more nuanced clinical judgement and led to the modest acceptance rates seen in the AMS group. One identified barrier to accepting recommendations was the involvement of multiple providers, particularly when care was delivered across different health systems. When antiplatelet therapy was managed by a cardiologist or other specialist outside our organization, AMS was unable to communicate directly through the electronic health record. Instead, alternative methods such as fax or telephone communication were utilized, which posed challenges to timely and effective transmission of recommendations. Our recommendations are informed by internal guidelines and protocols, which closely align with national guidelines that support deprescribing antiplatelets in patients with stable CAD after one year and adding a gastric prophylaxis agent for high-risk patients. However, some providers may be more cautious about adopting guideline recommendations to stop antiplatelets, particularly in patients with higher risk for recurrent cardiovascular events such as stent thrombosis. While national cardiovascular society guidelines recommend limiting dual antithrombotic therapy to one year in most patients, they also emphasize an individualized approach to antithrombotic management and advise shared decision-making with consideration of patient-specific bleeding and thrombotic risk. For patients with high ischemic risk, such as those with complex coronary lesions, extensive atherosclerosis, or recurrent ischemic events, continuation of antiplatelet may be justified especially when bleeding risk is low. Although we did not assess the clinical outcomes of ischemic or bleeding events, the increased bleeding risk associated with dual or triple therapy and the protective benefits of PPIs against UGIB are well-established in the literature [4–9, 15–23]. Furthermore, aspirin deprescribing initiatives have proven to reduce the rates of bleeding in anticoagulated patients [30].

This study has several limitations. First, the results of our study may be understated as medication use data for the non-AMS group were collected solely based on electronic medical records and were not confirmed through patient interviews, as was done in the study by Medor et al. [29]. Since antiplatelet use, particularly over-the-counter aspirin, was not confirmed in the non-AMS group, actual use may have been higher than reported, which could further increase the difference observed between the usual care and AMS groups. Second, this study only evaluated the impact of the annual review initiative when it was first launched in 2023, and the AMS group only included patients enrolled in AMS for at least one year prior to the end of the study period who underwent the 2023–2024 annual review. Patients who were enrolled in AMS after October 2023 had not yet received an annual review and were excluded, even though recommendations to stop unnecessary antiplatelets and initiate PPIs for UGIB prophylaxis could still have been made during enrollment or as part of routine AMS care. Additionally, we did not perform multivariable adjustment for baseline patient characteristics that may have influenced bleeding or thrombotic risk and patterns of antiplatelet or PPI use between groups. Nevertheless, such statistical modeling would not have impacted the outcomes related to the intervention, the annual review, which was only provided to the AMS group. Data regarding provider type, specialty, and relevant patient characteristics like history of bleeding or history of procedural interventions were not collected, which may have limited our ability to further characterize factors influencing acceptance of AMS recommendations. Additionally, patients were not followed longitudinally; therefore, adjustments to antiplatelet or PPI therapy may have occurred after the annual review due to changes in clinical status during the study period. As a result, the observed reduction in antiplatelet use and increase in PPI use at the end of the study represent net changes rather than absolute changes. Finally, this study was conducted among a cohort of patients from a single ambulatory care organization in Massachusetts and results may not be generalizable to a broader population.

## Conclusion

The AMS intervention was effective in lowering antiplatelet use among patients on DOAC and increasing prescribing rates of PPIs among patients on dual antithrombotic therapy compared to usual care. Despite modest acceptance rates of AMS recommendations during the intervention study period, the overall prescribing rates of antiplatelets and gastric bleeding prophylaxis highlight one of the many valuable roles a centralized AMS can have in optimizing antithrombotic therapy within a primary care setting.

This quality initiative is one of several systematic approaches employed by AMS to reduce bleeding and thrombotic events in patients taking DOACs. In addition, AMS responsibilities include maintaining up-to-date lab monitoring, ensuring timely dose adjustments, managing perioperative anticoagulation, providing patient education, addressing drug-drug interaction, facilitating transitions of care, monitoring adherence, and selecting cost-effective, clinically appropriate anticoagulant agents. Further research is needed to confirm the effectiveness of these initiatives in preventing bleeding while preserving cardiovascular protection. Additionally, future investigations should explore barriers to prescriber adoption of AMS recommendations, particularly in light of the modest acceptance rates observed in this study.

## Supplementary Information

Below is the link to the electronic supplementary material.

Supplementary Material 1

## Data Availability

No datasets were generated or analysed during the current study.
