# Supplementary material for: Impact of an Anticoagulation Management Service–Led Annual Review on Antiplatelet Use and Gastrointestinal Bleeding Prophylaxis in Ambulatory Patients on Background Direct Oral Anticoagulants
Source: J Thromb Thrombolysis. 2026 Apr 29;59(6):1600–10. doi: 10.1007/s11239-026-03283-7 (PMC13447394; doi:10.1007/s11239-026-03283-7)
Supplement: Supplementary file 1 [file 11239_2026_3283_MOESM1_ESM.docx]

**Supplementary Appendix**

**Introduction to Anticoagulation Management Service (AMS)**

The Atrius Health Anticoagulation Management Service (AMS) is a nurse and clinical pharmacist staffed program that provides comprehensive anticoagulation management services for all oral anticoagulants, including warfarin and direct oral anticoagulants (DOACs). The service supports Atrius Health primary care and specialty clinicians by providing patient education and overseeing anticoagulation therapy.

AMS is structured to promote adherence to evidence-based guidelines by centralizing responsibility for patient monitoring and anticoagulant therapy adjustments in accordance with departmental clinical guidelines, practice protocols, and the thrombosis collaborative drug therapy management (CDTM) protocol.

The service is available to all patients receiving primary care through Atrius Health Internal Medicine practices and requires referral from a primary care physician (PCP) or collaborating prescribing physician within Atrius Health. At the time of referral, the PCP or collaborating physician establishes the anticoagulation treatment plan, including the indication, treatment goals, and duration of therapy. After enrollment and patient consent, AMS assumes responsibility for day-to-day anticoagulation management, including coordination of periprocedural therapy interruptions and necessary treatment adjustments. Any deviations from established protocols require approval from a clinical pharmacy consultant or a member of the patient’s physician team.

**Annual Patient Review**

Patients receiving long-term anticoagulation should complete a therapy review at least annually to assess the need for continued anticoagulation and the appropriateness of the individualized antithrombotic treatment plan. This review includes assessment of the indication and planned duration of therapy, selection and dosing of the anticoagulant agent, quality of anticoagulation control (e.g., warfarin time in therapeutic range or adherence to direct oral anticoagulants), concurrent antiplatelet therapy, and the patient’s thrombotic and bleeding risk.

For antithrombotic plans that require collaboration with the referring physician and/or responsible care team to facilitate shared decision-making, for patients needing reassessment of therapy duration or those with complex or atypical regimens, AMS staff will update summary and detailed documentation supporting any recommended therapy modifications in a progress note, which is forwarded to the referring physician or responsible care team. Such clinical scenarios could include patients receiving concomitant anticoagulant and antiplatelet therapy, with the exception of individuals with mechanical valve replacement, those treated with rivaroxaban 2.5 mg twice daily plus aspirin 81 mg daily for prevention of major cardiovascular events in coronary artery disease or peripheral artery disease, and those with peripheral artery disease requiring vascular intervention.

If patients receive care from physicians outside of Atrius, AMS is unable to communicate directly through the internal electronic health record. In such cases, AMS communicates recommendations using alternative methods, including telephone calls or fax correspondence to the external physician’s office.

**Supplementary Figure 1**. AMS Annual Review Workflow

Patients enrolled in AMS for at least 1 year

Patients received annual review and antithrombotic therapy assessment from October 1, 2023 to September 30, 2024.

Patients with optimized antithrombotic therapy and no therapy changes needed.

1. Patient continue current therapy. 2. AMS staff document annual review completion.

Patients needing antithrombotic therapy reassessment, including concomittant anticoagulant and antiplatelet use

AMS staff document annual review completion and provide recommendation for therapy changes and supporting evidence based on AMS guidelines and practice protocol.

Message is sent to provider(s) and involved care team(s). If patient has external provider, recommendations will be communicated via phone or fax.

Provider(s) accept recommendation and implement changes.

Provider(s) decline and document reasons for declining (if available).

No response: AMS staff follow up after 4 weeks from initial message.

**AMS Protocols for Antiplatelet Management in Patients Requiring Indefinite Anticoagulation for Nonvalvular Atrial Fibrillation (AF) or Venous Thromboembolism** (**VTE)**

Anticoagulant Considerations:

- Use of an anticoagulant (AC) plus single antiplatelet therapy (SAPT) increases the risk of minor, major, intracranial, and life-threatening bleeding compared to anticoagulation alone. The addition of dual antiplatelet therapy (DAPT) to oral anticoagulation, known as triple antithrombotic therapy, further increases the risk of bleeding.
- For men with history of AF and CHADS2-VASc score 0 or 1 and women with AF and CHADS2-VASc score 1 or 2, reassess bleeding risk (e.g., HAS-BLED score) [1] and consider the risks and benefits of oral anticoagulation during the periods when SAPT or DAPT are needed.
- For patients on anticoagulation for VTE indications who have completed the acute VTE treatment phase, reassess bleeding risk (e.g., HAS-BLED score) [1] and consider the risks and benefits of oral anticoagulation in the setting of SAPT or DAPT.
- If indicated and feasible, DOACs are preferred over warfarin for oral anticoagulation in the setting of any antiplatelet therapy to minimize rates of major, intracranial, and fatal bleeding.
- If the patient is a candidate for a reduced dose of DOAC for extended prevention of recurrent VTE (rivaroxaban 10 mg once daily or apixaban 2.5 mg twice daily), this regimen is preferred in the setting of any concomitant antiplatelet therapy.

Strategies below apply only to patients anticoagulated for nonvalvular AF or VTE and do not include recommendations for utilizing low-dose DOACs (i.e., rivaroxaban 2.5mg twice daily) for secondary CVD prevention.

1. Primary prevention of atherosclerotic cardiovascular disease (ASCVD)

- There is no anticipated benefit to using antiplatelets for primary prevention of ASCVD in patients on anticoagulation.
- Given the risk of bleeding, antiplatelet therapy should always be discontinued at the time of anticoagulation initiation.

2. Chronic coronary disease*

- Bleeding risk may be assessed using scoring tools like HAS-BLED [1] or utilizing the Academic Research Consortium definition for high bleeding risk (ARC-HBR) [5] (see page 7, Bleeding Risk Score for more details).

**Supplementary Figure 2.** AMS Protocol on Antiplatelet strategies for chronic coronary disease for patients requiring anticoagulation

History of Acute Coronary Syndrome or Chronic coronary disease

Recent ACS (0-12 months after ACS +/- PCI) [2-4]

Up to 1 month post ACS: AC + P2Y12i + ASA continued until time of discharge from hospital or a few days longer, generally up to 1 week; use for up to 30 days is reasonable if patient is at high thrombotic risk and low bleeding risk

For 12 months post ACS: AC + P2Y12i. If bleeding risk is high, discontinuation of P2Y12i at 6 months may be reasonable after weighing relative risks of stent thrombosis vs bleeding risk

> 12 months post ACS: AC monotherapy. If thrombotic/ischemic risk is high and bleeding risk is low, continuation of AC + SAPT beyond 12 months may be reasonable in select patients

Recent Elective PCI (0-12 months after stent placement) [2]

Up to 1 month post PCI: AC + P2Y12i + ASA continued until time of discharge from hospital or a few days longer, generally up to 1 week; use for up to 30 days is reasonable if patient is at high thrombotic risk and low bleeding risk

For 6 months (1 month if BMS) post PCI: AC + P2Y12i. If bleeding risk is high, discontinuation of P2Y12i at 3 months may be reasonable after weighing relative risks of stent thrombosis vs bleeding risk

For 6 (1 if BMS) to 12 months post PCI: AC + SAPT (P2Y12i or ASA)

12 months post PCI: AC monotherapy. If thrombotic/ischemic risk is high and bleeding risk is low, continuation of AC + SAPT beyond 12 months may be reasonable in select patients

CCD (> 12 months after ACS or PCI or no history of ACS/PCI) [2]

AC monotherapy. If thrombotic/ischemic risk is high and bleeding risk is low, continuation of AC + SAPT beyond 12 months post ACS/PCI may be reasonable in select patients

CCD s/p CABG and no history of ACS [2]

If <12 months from CABG: AC + ASA <100mg/day

If >12 months from CABG: AC monotherapy

ACS acute coronary syndrome, PCI percutaneous coronary intervention, AC anticoagulant (e.g., DOAC, warfarin, low molecular weight heparin), P2Y12i P2Y12 inhibitor (clopidogrel, ticagrelor, or prasugrel), ASA aspirin, SAPT single antiplatelet therapy, PCI percutaneous coronary intervention, BMS bare metal stent, CABG coronary artery bypass grafting, CCD chronic coronary disease

*High thrombotic risk (early events): ACS, previous stent thrombosis while on antiplatelet treatment, PCI complexity (3 vessels treated, ≥3 stents implanted, ≥3 lesions treated, bifurcation with 2 stents implanted, total stent length >60 mm, surgical bypass graft PCI, chronic total occlusion PCI, atherectomy device use, left main PCI) [3]; High ischemic risk (long-term events): previous myocardial infarction, multivessel coronary artery disease, polyvascular disease, diabetes mellitus, chronic kidney disease, heart failure^2^

3. History of cerebrovascular disease

**Supplementary Figure 3.** AMS Protocol on Antiplatelet strategies for cerebrovascular disease for patients requiring anticoagulation

History of Cerebrovascular Disease

History of CVA/TIA [2]

AC monotherapy

History of carotid endarterectomy [2]

AC monotherapy

History of carotid stenting [2]

1-3 months (or for standard duration of DAPT) post stenting: AC + P2Y12i

> 3 months (or after standard duration of DAPT has ended) post stenting: AC monotherapy

CVA cerebrovascular accident, TIA transient ischemic attack, AC anticoagulant (e.g., DOAC, warfarin, low molecular weight heparin), DAPT dual antiplatelet therapy, P2Y12i P2Y12 inhibitor (clopidogrel, ticagrelor, or prasugrel)

4. Peripheral artery disease

**Supplementary Figure 4.** AMS Protocol on Antiplatelet strategies for peripheral artery disease for patients requiring anticoagulation

Peripheral Artery Disease

No prior intervention or surgical repair [2]

AC monotherapy

History of endovascular intervention/stenting [2]

1-3 months (or for standard duration of DAPT) post stenting: AC + P2Y12i

> 3 months (or after standard duration of DAPT has ended) post stenting: AC monotherapy

AC anticoagulant (e.g., DOAC, warfarin, LMWH (in setting of VTE)), DAPT dual antiplatelet therapy, P2Y12i P2Y12 inhibitor (clopidogrel, ticagrelor, or prasugrel)

**AMS Protocol for** **Bleeding Risk Score**

Bleeding risk may be assessed using scoring tools like HAS-BLED [1] or utilizing the Academic Research Consortium definition for high bleeding risk (ARC-HBR) [5].

1. HAS-BLED

At Atrius Health AMS, we are now using HAS-BLED to determine bleeding risk scores of our patients, the relevant risk factors are noted below. Bear in mind that there are other available bleeding risk scores, which may consider some factors not in the HAS-BLED tool.

HAS-BLED 0-2 = low bleed risk: HAS-BLED ≥3 = high bleed risk: HAS-BLED ≥3 plus history of major bleeding = unusually high bleeding risk

**Supplementary Table 1.** HAS-BLED score

| **Bleeding risk score** | **Points** |
| --- | --- |
| Hypertension History (uncontrolled, >160 systolic) | 1 |
| Renal disease (Dialysis, transplant, serum creatine >2.26 mg/dL) | 1 |
| Liver disease (Cirrhosis or Bilirubin >2x Normal or AST/ALT/AP >3x Normal)* | 1 |
| History of Stroke | 1 |
| Prior Major Bleeding or Predisposition to Bleeding | 1 |
| Labile International Normalized Ratio (INR) on warfarin | 1 |
| Age > 65 years | 1 |
| Medication Usage Predisposing to Bleeding (nonsteroidal anti-inflammatory drugs (NSAIDs), antiplatelet agents) | 1 |
| Alcohol or Drug Usage history (≥8 drinks/week) | 1 |

* AST aspartate aminotransferase, ALT alanine aminotransferase, AP alkaline phosphatase

2. Academic Research Consortium for High Bleeding Risk (ARC-HBR)

High bleeding risk (HBR) in patients undergoing PCI defined by the Academic Research Consortium for High Bleeding Risk (ARC-HBR) [5].

Patients are considered to be at high bleeding risk if at least 1 major or 2 minor criteria are met:

**Supplementary Table 2.** Academic Research Consortium High Bleeding Risk Criteria After PCI

| **Major criteria** | **Minor criteria** |
| --- | --- |
| - Anticipated use of long-term oral anticoagulation (excluding vascular protection doses) - Severe or end-stage chronic kidney disease (CKD) (eGFR <30 mL/min) - Hemoglobin <11 g/dL - Spontaneous bleeding requiring hospitalization or transfusion in the past 6 months or at any time, if recurrent - Moderate or severe baseline thrombocytopenia (platelet count <100×10^9^/L) - Chronic bleeding diathesis - Liver cirrhosis with portal hypertension - Active malignancy (excluding nonmelanoma skin cancer) within the past 12 months - Previous spontaneous intracerebral hemorrhage (ICH) at any time - Previous traumatic ICH within the past 12 months - Presence of a brain arteriovenous malformation (bAVM) - Moderate or severe ischemic stroke (National Institutes of Health Stroke Scale (NIHSS) ≥5) within the past 6 months - Nondeferrable major surgery on DAPT - Recent major surgery or major trauma within 30 days before PCI | - Age ≥75 years - Moderate CKD (eGFR 30 to 59 mL/min) - Hemoglobin 11 to 12.9 g/dL for men and 11 to 11.9 g/dL for women - Spontaneous bleeding requiring hospitalization or transfusion within the past 12 months not meeting the major criterion - Long-term use of oral nonsteroidal anti-inflammatory drugs (NSAIDs) or steroids - Any ischemic stroke at any time not meeting the major criterion |

**AMS Protocol for Gastrointestinal Bleeding Prophylaxis**

We recommended addition of proton pump inhibitor (PPI) or histamine-2 receptor antagonist (H2RA) if PPI is contraindicated in patients at high risk of bleeding, including patients treated with two antithrombotic agents or history of gastrointestinal (GI) bleeding. One exclusion to this is patients taking rivaroxaban 2.5 mg twice daily with aspirin 81 mg daily for the reduction of major cardiovascular events in CAD or major thrombotic vascular events in peripheral artery disease. PPI use in this population has not been shown to reduce GI bleeding risk. In other patients with increased risk of GI bleeding, including those with advanced age and those with concomitant use of warfarin, steroids, or NSAIDs use of a PPI is reasonable. Concomitant administration of aspirin and nonselective NSAIDs, such as ibuprofen, should be avoided if possible to reduce the risk of GI bleeding. Routine use of PPIs is not recommended for patients at low risk of GI bleeding [6].

There have been concerns regarding reduced efficacy of clopidogrel with concomitant PPI use (particularly omeprazole). However, in the only randomized controlled trial on this topic, omeprazole was protective for GI bleeding without an increase in ischemic events [7].

**References**

1. Pisters R, Lane DA, Nieuwlaat R, de Vos CB, Crijns HJ, Lip GY. A novel user-friendly score (HAS-BLED) to assess 1-year risk of major bleeding in patients with atrial fibrillation: the Euro Heart Survey. Chest. 2010;138(5):1093-1100.
2. Kumbhani DJ, Cannon CP, Beavers CJ, et al. 2020 ACC Expert Consensus Decision Pathway for Anticoagulant and Antiplatelet Therapy in Patients With Atrial Fibrillation or Venous Thromboembolism Undergoing Percutaneous Coronary Intervention or With Atherosclerotic Cardiovascular Disease: A Report of the American College of Cardiology Solution Set Oversight Committee. J Am Coll Cardiol. 2021;77(5):629-658.
3. Angiolillo DJ, Bhatt DL, Cannon CP, et al. Antithrombotic Therapy in Patients With Atrial Fibrillation Treated With Oral Anticoagulation Undergoing Percutaneous Coronary Intervention: A North American Perspective: 2021 Update. Circulation. 2021 Feb 9;143(6):583-596.
4. Rao SV, O'Donoghue ML, Ruel M, et al. 2025 ACC/AHA/ACEP/NAEMSP/SCAI Guideline for the Management of Patients With Acute Coronary Syndromes: A Report of the American College of Cardiology/American Heart Association Joint Committee on Clinical Practice Guidelines. Circulation. 2025;151(13):e771-e862.
5. Urban P, Mehran R, Colleran R, et al. Defining High Bleeding Risk in Patients Undergoing Percutaneous Coronary Intervention. Circulation. 2019;140(3):240-261.
6. Levine GN, Bates ER, Bittl JA, et al. 2016 ACC/AHA guideline Focused Update on duration of Dual Antiplatelet therapy in patients with coronary artery disease. J Am Coll Cardiol. 2016;68(10):1082.
7. Bhatt DL, Cryer BL, Contant CF, et al. Clopidogrel with or without omeprazole in coronary artery disease. N Engl J Med. 2010;363(20):1909-1917.
